# The London Hospitals of Earlier Days

**Published:** 1913-03-29

**Authors:** Frederic Bullen


### MEDICAL ANTIQUITIES.

### The London Hospitals of Earlier Days.

By FREDERIC BULLEN.

ONE day, probably, the history and evolution of the London hospitals will be written by a competent person. In the meantime I am venturing to pen a few words, collected from various sources, concerning two or three of our most ancient and important institutions and their constitution in the early eighteenth century. Far back as this may seem, we can find the existence of what is now one of the wonders of the civilised world—namely, voluntary organisations providing the best and most advanced medical assistance. It is impossible to estimate what is owing to these institutions, and in these works of almost two hundred years ago we can see the finger-post pointing to the high road upon which our medical charities are now travelling. To my mind the change from gross superstition to real science was most marked at this period; and, judging from those hospital pharmacopœias which survive, it was hospital practice which helped the forward movement.

Chamberlayne's "Magnæ Britanniæ Notitia," which was a veritable "Whitaker" and crowded full of information, gives some interesting facts concerning the hospitals in London. The volume for 1727 tells us that the settlement of allowances for charitable institutions dates from the Reformation; previous to this the religious bodies had undertaken the work. In 1553 Dr. Ridley, then Bishop of London, preached a remarkable sermon upon the subject of Charity before King Edward VI., which moved that young monarch to form a Commission to consider the entire question of London Charities. As an outcome the King set by the Hospitals of St. Bartholomew and St. Thomas for the provision of the sick and wounded. St. Bartholomew's Hospital had been founded by the Greyfriars in the period of Henry I. upon its present site. It had escaped burning during the Fire of London, but most of its estate had been consumed; and we are told that "here are sometimes 2,000 or upwards cured in a year, and relieved with monies and other necessaries at their departure."

St. Thomas's Hospital, situate then in Southwark, was founded in 1213 by the Prior of Bermondsey; and, being surrendered to King Henry VIII. during the Reformation, it was valued at £266 per annum. As above, it was given to the Mayor of London for public use by King Edward VI., and was ordered to be called the Hospital of St. Thomas the Apostle. It escaped burning in 1666, 1676, 1681, and in 1689. In this year of 1727 it is described as consisting of "four Courts, very spacious, ornamental, and commodious. In the first are six Wards for Women. In the second are two Chapels, one for the use of the Hospital, the other commonly called The Parish Church of St. Thomas, Southwark. In the same Court are the houses of the Treasurer, Hospitaller, Steward, Butler, and Cook. In the third Court are seven Wards for Men, with a convenient Shop, Store-rooms, and Laboratory for the Apothecary. In the fourth Court are two Wards for Women, with the Surgery, hot and cold baths, &c. Besides these Squares, the Governors, in 1718, erected a spacious Building, in which are Wards and Beds in them for one hundred persons, so that now there is room for above five hundred people, and there are about four thousand poor and diseased persons cured in and discharged yearly out of this Hospital." An important note then follows in these words: "On a convenient Piece of Ground in the Parish of St. Thomas, Southwark, near adjoining to this Hospital, is now laid the foundations of an *Hospital For Incurables*, by the unparalleled charity of Thomas Guy, Esq., in which there will be provision made for about four hundred persons." The staff at St. Thomas's Hospital included a chaplain, three physicians, four surgeons, an apothecary, one cook, one butler ("who hath also charge of the Brew-house"), two porters, four beadles, one matron, sixteen sisters, with nurses, helpers, and watchers. The Treasurer had the executive government, with a committee of thirty governors and twelve almoners. At St. Bartholomew's there was a chaplain, two physicians, an apothecary, three surgeons, three assistant surgeons, two "surgeons for cutting for the stone," and a surgeon and chaplain each for the Kingsland Hospital and the Lock Hospital (then situate in Southwark), which were both under the charge of the larger institution. Later, in 1755, Chamberlayne enumerates the staffs also of St. George's Hospital, Hyde Park Corner, the Infirmary in St. James's Street, Westminster (instituted in 1719), Guy's Hospital, and the London Infirmary, in Prescot Street, Goodman's Fields. The equipment for Guy's included a "surgery-man" and a "Keeper of the Lunatics." St. George's Hospital had a lengthy and distinguished list of honorary officers, including seven "Apothecaries who attend by rotation, as visitors to see the House Apothecaries, take due care of the Patients and Medicines, and that they are dispensed according to the prescriptions of the physicians." The physicians visited three times a week and the surgeon attended daily.

Curious reading is the diet chart of the hospitals at this time, although one feels that the patients may have found them uninteresting. "The Modern Practice of the London Hospitals" (1744), which deals with St. Thomas's, St. Bartholomew's, St. George's, and Guy's Hospitals, gives the particulars. A Full Diet, Low Diet, Milk Diet, Fish Diet, Dry Diet, Raisin Diet, and a Diet for patients under salivation are given. Here is the "Full Diet"!

| — | Sun. & Thurs. | Tues. & Sat. | Mon. & Fri. | Wed. |
|---|---|---|---|---|
| Breakfast | Pint of water gruel | Pint of water gruel | Pint of milk pottage | Pint of milk pottage |
| Dinner | ½ lb. boiled beef with greens | ½ lb. boiled mutton with greens | A pint of rice, milk or plumb broth | ½ lb. boiled pudding |
| Supper | A pint of broth | A pint of broth | 2 oz. of cheese or butter | A pint of water gruel |

The patients upon Full Diet had one loaf of bread (14 ounces) per day, three pints of small beer daily from Lady Day to Michaelmas, and a quart daily from Michaelmas to Lady Day. Patients on Fish Diet "shall have Fish for Dinner on Mondays, Wednesdays, and Fridays, if it can conveniently be had; if not, the Low Diet." Patients upon the Raisin Diet "shall have half a pound of Raisins per day, as much Bread as they can eat, a quart of the strong decoction of Guaiacum, and as much of the weak decoction as they can drink."

A page out of THE HOSPITAL to-day, with its articles and discussions upon hospital diets, would have turned an eighteenth-century hospital patient speechless with envy. As I have said the hospital pharmacopœias show the shaking off of the older unscientific methods. From the St. George's Hospital came this prescription for stone in the bladder: "Take calcined oyster-shells a pound and pour on them a gallon of boiling water, the water should stand four hours or longer on the shells, and it should be made in an earthern vessel." This, according to the eminent Dr. Whytt, possessed all the lithontriptic power of the famous Mrs. Stephen's nostrum, which was purchased by Parliament for £5,000, and which proved to be composed of calcined snails! In the St. Thomas's Hospital section we read that the dose of the Lambeth Wells water was half a pint three times a day, and the Sydenham Wells water figures in a formula. Millepedes and snails were still in hospital use according to this book and to Henry Banyer, author of "The Pharmacopœia Pauperum" (1718). In the latter book this former pupil of St. Thomas's reproduces famous prescriptions of Dr. Richard Mead and his contemporaries. However, these snails, etc., are almost the only unpleasant things in the hospital formulas; and this marks an advance æsthetically if in no other way. For, after all, the physician of that day, when ordering a consumptive patient to gather snails and earthworms early in the morning, for internal administration, was putting that patient upon fresh air and fattening food, which only finds a successor to-day in sanatorium and cod-liver oil. From discovering what articles relieved certain conditions the physicians commenced to learn why, and were then able to replace the crabs' claws, urine, etc., of their prescriptions by the chemical salts which were then being produced. And to-day we are still endeavouring to replace animal products by synthetically produced chemical products. With Walt Whitman the practitioner and hospital administrator can say: "Whither I walk I cannot define, but I know it is good; the past and present indicate that it is good."

